# Webbasierte Genexpressionsanalysen – auf dem Weg zur molekularen Entschlüsselung gesunder und erkrankter Augengewebe

**DOI:** 10.1007/s00347-022-01592-9

**Published:** 2022-02-22

**Authors:** Julian Wolf, Thabo Lapp, Thomas Reinhard, Hansjürgen Agostini, Günther Schlunck, Clemens Lange

**Affiliations:** 1 Klinik für Augenheilkunde, Universitätsklinikum Freiburg, Medizinische Fakultät, Universität Freiburg, Freiburg, Deutschland; 2 Ophtha-Lab, Department of Ophthalmology, St. Franziskus Hospital, Muenster, Muenster, Deutschland

**Keywords:** Transkriptom, RNA-Sequenzierung, Datenbank, Auge, Biomarker, Transcriptome, RNA-Seq, Database, Eye, Biomarker

## Abstract

**Hintergrund:**

Die Entschlüsselung des Transkriptoms hat in den letzten Jahren unser Verständnis zahlreicher Erkrankungen verbessert. Öffentlich zugängliche Datenbanken, wie z. B. die *Gene Expression Omnibus*-Datenbank des *National Center for Biotechnology Information*, sammeln Transkriptomrohdaten aus einer Vielfalt von Proben, ohne jedoch dem bioinformatischen Laien einen intuitiven Zugang zu den Daten zu gewähren. Daher wurden in den vergangenen Jahren spezielle Transkriptomdatenbanken programmiert, die eine benutzerfreundliche Web-basierte Datenanalyse ermöglichen und damit niederschwellig molekulare Einblicke in okuläre Gewebe ermöglichen.

**Fragestellung:**

Ziel dieser Arbeit ist es, einen Überblick über die aktuell verfügbaren okulären Transkriptomdatenbanken zu geben und diese mit dem in Freiburg neu etablierten *Human Eye Transcriptome Atlas* zu vergleichen.

**Methoden:**

Literatursuche in PubMed.

**Ergebnisse:**

Neun okuläre Transkriptomdatenbanken mit unterschiedlichem Anwendungsschwerpunkt wurden identifiziert. Die Plattformen *iSyTE* und *Express *spezialisieren sich auf die Genexpression während der Linsen- und Netzhautentwicklung der Maus, wohingegen *retina.tigem.it, Eye in a Disk* und *Spectacle* ihren Fokus auf einzelne okuläre Gewebe wie die Netzhaut legen. *Spectacle, UCSC Cell Browser *und *Single Cell Portal *erlauben die intuitive Exploration von Einzelzell-RNA-Sequenzierungsdaten von Netzhaut‑, Aderhaut‑, Kornea‑, Iris‑, Trabekelmaschenwerk- und Skleragewebe. Die Microarray-Profile verschiedener gesunder okulärer Gewebe werden in der *Ocular Tissue Database *bereitgestellt. Der *Human Eye Transcriptome Atlas* erfasst derzeit die größte Vielfalt an Augengeweben und Erkrankungen des Auges. Er zeichnet sich durch einen hohen Qualitätsstandard aus, der durch methodische Homogenität erreicht wird.

**Schlussfolgerungen:**

Okuläre Transkriptomdatenbanken bieten einen umfassenden und intuitiven Einblick in die Transkriptionsprofile verschiedener gesunder und erkrankter Augengewebe. So verbessern sie unser Verständnis der zugrunde liegenden molekularen Krankheitsprozesse, unterstützen die Hypothesengenerierung und helfen bei der Suche nach neuen diagnostischen und therapeutischen Zielen für verschiedene Augenerkrankungen.

## Abstract (en)

**Background:**

Gene expression analysis using RNA sequencing has helped to improve the understanding of many diseases. Databases, such as the *Gene Expression Omnibus database* of the *National Center for Biotechnology Information* provide RNA sequencing raw data from various diseased tissue types but their analysis requires advanced bioinformatics skills. Therefore, specific ocular databases provide the transcriptional profiles of different ocular tissues and in addition enable intuitive web-based data analysis.

**Objective:**

The aim of this narrative review is to provide an overview of ocular transcriptome databases and to compare them with the *Human Eye Transcriptome Atlas* newly established in Freiburg.

**Methods:**

PubMed literature search.

**Results:**

A total of nine ocular transcriptome databases focusing on different aspects were identified. The *iSyTE* and *Express* platforms specialize in gene expression during lens and retinal development in mice, whereas *retina.tigem.it*, *Eye in a Disk*, and *Spectacle* focus on selected ocular tissues such as the retina. *Spectacle*, *UCSC Cell Browser* and *Single Cell Portal* allow intuitive exploration of single cell RNA sequencing data derived from retinal, choroid, cornea, iris, trabecular meshwork and sclera specimens. The microarray profiles of a variety of healthy ocular tissues are included in the *Ocular Tissue Database*. The *Human Eye Transcriptome Atlas* provides the largest collection of different ocular tissue types, contains the highest number of ocular diseases and is characterized by a high level of quality achieved by methodological consistency.

**Conclusion:**

Ocular transcriptome databases provide comprehensive and intuitive insights into the transcriptional profiles of a variety of healthy and diseased ocular tissues. Thus, they improve our understanding of the underlying molecular mediators, support hypothesis generation and help in the search for new diagnostic and therapeutic targets for various ocular diseases.

Next Generation Sequencing (NGS) ermöglicht die simultane Sequenzierung von Millionen DNA- oder RNA-Molekülen und hat in den vergangenen Jahren unzählige Forschungsfelder wie die biologische Grundlagenforschung und die Untersuchung krankheitsrelevanter Prozesse revolutioniert. Während das Genom die Informationen der DNA beschreibt, die in allen Zellen identisch ist, spiegelt das Transkriptom die Gesamtheit aller RNA-Moleküle wider und ist somit dynamisch und in verschiedenen Zellen und Geweben unterschiedlich ausgeprägt. Die Transkriptomanalyse mittels RNA-Sequenzierung nimmt somit eine besondere Rolle ein, um den Funktionszustand eines Gewebes zu erfassen und findet in der Klinik zunehmend Anwendung, z. B. bei der diagnostischen Klassifikation von Tumoren [9], der Abschätzung der Tumorprognose [28] sowie bei der Vorhersage des Therapieansprechens [7]. Große Datenbanken wie der *Cancer Genome Atlas* [6] stellen die in wissenschaftlichen Arbeiten generierten Sequenzierungsrohdaten zur Verfügung, wobei bisher kaum Augengewebe enthalten ist und die Analyse der Rohdaten bioinformatische Kenntnisse erfordert. Daher wurden in den vergangenen Jahren spezielle benutzerfreundliche und web-basierte Datenbanken erstellt, die eine intuitive Durchsicht und vergleichende Analyse der Transkriptionsprofile okulärer Gewebe ermöglichen. Das Ziel dieser Arbeit ist es, dem Leser einen Überblick über diese aktuell verfügbaren okulären Transkriptomdatenbanken zu geben und deren Vorteile bzw. Limitation aufzuzeigen.

## Prinzip der RNA-Sequenzierung

**Figure 1:**
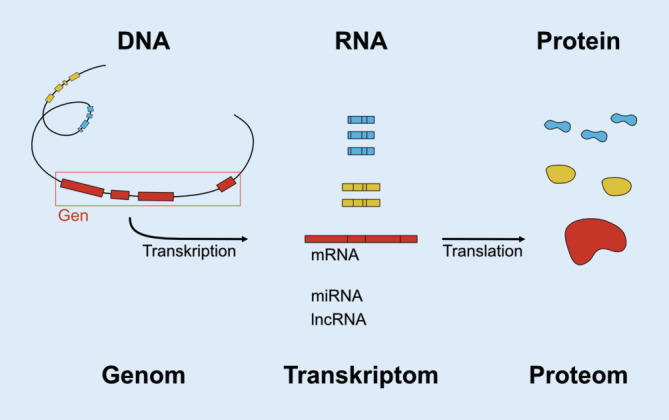

Die RNA-Sequenzierung erlaubt die Entschlüsselung der Nukleotidsequenzen von Millionen von RNA-Molekülen in einer Probe [24]. Durch Vergleich dieser Sequenzen mit dem bekannten Referenzgenom können unterschiedliche RNA-Moleküle identifiziert und quantifiziert werden. Die RNA dient unter anderem als Bauplan für die Herstellung von Proteinen oder kann regulatorische Funktionen in diesem Prozess ausüben. Die Analyse des Transkriptoms bietet somit einen unvoreingenommenen Einblick in den Funktionszustand des Gewebes (Abb. 1).

Der Vergleich von Proben erkrankter und gesunder Probanden ermöglicht genaue Einblicke in die der Erkrankung zugrunde liegenden pathophysiologischen Prozesse und darüber hinaus die Identifikation neuer diagnostischer und prognostisch relevanter Biomarker. Der Erfolg des *Human Genome Projects* [13] und die technischen Fortschritte haben in den letzten Jahren die Kosten und den Zeitaufwand der Sequenzierung erheblich reduziert, und es ist wahrscheinlich, dass diese Entwicklung zu einer zunehmenden Anwendung der Technologie in der klinischen Diagnostik führen wird [8]. Zudem können neuerdings durch spezielle Sequenzierungsmethoden neben frischen Proben auch archivierte Formalin-fixierte und in Paraffin eingebettete Präparate sequenziert werden, was insbesondere die Analyse seltener Erkrankungen deutlich vereinfacht [2].

## Anwendung in der Onkologie

Die Transkriptomanalyse hat bisher insbesondere Anwendung in der Onkologie gefunden [7, 9, 28]. So wurden anhand von Transkriptomdaten von Lungentumoren und Kontrollgewebe diagnostische Faktoren identifiziert, auf deren Basis in einem unabhängigen Validierungsdatensatz aus über 1000 Tumoren mit einer Genauigkeit von 98 % zwischen Tumor- und Kontrollgewebe unterschieden werden konnte [9]. Auch die Differenzierung von Plattenepithel- und Adenokarzinomen der Lunge gelang in dieser Arbeit mit einer Klassifikationsgenauigkeit von 95 % [9]. Ein weiteres Anwendungsbeispiel der RNA-Sequenzierung stellt die Abschätzung der Tumorprognose anhand des Transkriptionsprofils dar. Uhlen et al. analysierten das Transkriptom von über 8000 Proben der häufigsten Tumorarten und identifizierten für jede Entität prognostisch relevante Faktoren, anhand derer eine Prognoseabschätzung gelang [28]. Auch die Vorhersage des Therapieansprechens von Tumoren stellt eine interessante und ausgesprochen praxisrelevante Anwendungsmöglichkeit der Transkriptomanalyse dar. Die molekulare Charakterisierung verschiedener Tumoren mittels RNA- und DNA-Sequenzierung ermöglichte so eine Entitäten-übergreifende Klassifikation in 4 molekulare Subtypen, welche einen prädiktiven Wert für das Ansprechen auf eine Immuncheckpoint-Inhibitor-Therapie liefert und somit Relevanz für eine zukünftige individualisierte Therapieplanung hat [7]. Eine kürzlich veröffentlichte Stellungnahme der Bundesärztekammer geht davon aus, dass in den nächsten Jahren bei einem Großteil der Tumorpatienten schon bei Erstdiagnose eine molekulare Klassifikation des Tumors erfolgt mit dem Ziel, eine möglichst präzise Therapiestrategie zu verfolgen [20].

## Anwendung in der Augenheilkunde

In der Augenheilkunde hat die RNA-Sequenzierung insbesondere in der klinischen Praxis bisher vergleichsweise wenig Anwendung gefunden. Kürzlich wurde eine diagnostische Klassifikation von Plattenepithelkarzinomen und -Papillomen der Bindehaut anhand eines aus wenigen Markern bestehenden Transkriptionsprofils beschrieben [3, 15]. Zudem wurde die Genexpression von bestimmten Zellrezeptoren, die eine SARS-CoV-2-Infektion vermitteln, in Gewebe der Augenoberfläche [14] und intraokularen Geweben [16] mittels RNA-Sequenzierung untersucht. Auch Hyalozyten aus dem Glaskörper von Patienten mit epiretinaler Gliose konnten kürzlich mithilfe von RNA-Sequenzierungen als eine aktive und immunmodulatorische Zellpopulation charakterisiert werden [4]. Eine Prognoseabschätzung für okuläre Tumoren gelang für das Aderhaut- und das Bindehautmelanom [21, 32]. Das Aderhautmelanom konnte anhand des Transkriptoms in 4 prognostisch relevante molekulare Subtypen unterteilt werden [21]. Diese Klassifikation erreichte eine höhere Vorhersagekraft für das Auftreten von Fernmetastasen 5 Jahre nach einer Brachytherapie als die klassische Einteilung nach dem *American Joint Committee on Cancer Staging Manual (8th Edition) *[17]. Auch für das Bindehautmelanom wurden 20 prognostisch relevante Faktoren identifiziert, die eine Abschätzung des Risikos für das Auftreten eines Lokalrezidivs oder von Fernmetastasen ermöglichen [32]. Für die neovaskuläre altersabhängige Makuladegeneration (nAMD) wurden durch RNA-Sequenzierung von chorioidalen Neovaskularisationsmembranen (CNV) Calprotectin (*S100A8*/*S100A9*) sowie Secreted Phosphoprotein 1 (*SPP1*) als neue nAMD-assoziierte Faktoren identifiziert [22, 23, 31]. Die intravitreale Injektion eines SPP1-Inhibitors führte im murinen Laser-CNV-Modell zu einer signifikanten Modulation der CNV-Ausprägung, was die Bedeutung des Faktors als potenzielles neues Therapieziel für die nAMD unterstreicht [23].

## Transkriptomdatenbanken

Mit dem technischen Fortschritt, der zu einer erheblichen Zunahme von Transkriptomanalysen geführt hat, sind innerhalb der letzten Jahre große Datenbanken entstanden, die eine Vielzahl von Transkriptomdatensätzen verschiedener Erkrankungen enthalten [6, 10]. Eine der größten Datenbanken ist der *Cancer Genome Atlas*, welcher inzwischen die Sequenzierdaten von über 84.000 Tumorproben von 67 verschiedenen Entitäten enthält [6]. Die Vielfalt dieser Daten hat es ermöglicht, typische genetische und molekulare Veränderungen, die in verschiedenen Tumoren auftreten, zu katalogisieren, um zum einen das Wissen über jede einzelne Tumorentität zu erweitern und zum anderen das Verständnis über Entitäten-übergreifende Mechanismen der Karzinogenese zu verbessern [11]. Zudem sind die Sequenzierungsrohdaten öffentlich verfügbar und können z. B. als Validierungsdatensatz verwendet werden [9]. An dieser Stelle soll auch auf den *Human Protein Atlas *hingewiesen werden [27], der mithilfe einer Kombination verschiedener „Omics“-Technologien wie Massenspektrometrie oder antikörperbasierte Proteomik humane Proteine in Zellen, Geweben und Organen katalogisiert. Ungeachtet der genannten vielfältigen Möglichkeiten enthält der *Cancer Genome Atlas *mit Ausnahme des Aderhautmelanoms bisher keine okulären Gewebe. Obwohl effiziente Algorithmen für die Analyse der enthaltenen Sequenzierungsrohdaten existieren, erfordern diese spezielle bioinformatische Kenntnisse und sind darüber hinaus relativ zeitaufwendig. Aus diesen Gründen besteht ein Bedarf an Datenbanken, die Transkriptionsprofile okulärer Gewebe enthalten und gleichzeitig eine intuitive Datenanalyse ermöglichen.

## Übersicht okuläre Transkriptomdatenbanken

Nachfolgend wird eine Übersicht über die verfügbaren okulären Transkriptomdatenbanken gegeben (s. Tab. 1).

**Table 1:** 

Datenbank	*iSyTE 2.0*	*Express*	*Retina.tigem.it*	*Spectacle*	*UCSC Cell* *Browser*	*Broad Institute* *Single Cell* *Portal*	*Eye in a Disk*	*Ocular Tissue Database*	*Human Eye Transcriptome Atlas*
Gesundes Gewebe	Linse	Linse Retina	Retina	Retina RPE/Choroidea	Retina RPE/Choroidea Kornea	Kornea Iris TMW Sklera Retina RPE/Choroidea	Kornea Linse Retina RPE/Choroidea	Kornea Sklera TMW Iris Ziliarkörper Linse Sehnervenkopf Sehnerv Retina RPE/Choroidea	Bindehaut Kornea Lid Tränendrüse Sehnerv Periphere Retina Zentrale Retina RPE/Choroidea ILM Retinale Mikroglia Hyalozyten
Erkranktes Gewebe	–	–	–	Autoimmunretinopathie RPE nAMD	RPE nAMD	–	Retina AMD	–	BH-SCC BH-Papillom BH-Melanom Pterygium CNV-Membran Epiretinale Gliose PDR-Membran PVR epiretinal PVR subretinal
Gewebearten	1	2	1	4	4	6	5	10	20
Proben	42	56	50	23	23	18	829	6	139
Spezies	Maus	Maus	Mensch	Mensch	Mensch	Mensch, Schwein	Mensch	Mensch	Mensch
Gewebequelle	Maus	Maus	Postmortal	Postmortal	Postmortal	Postmortal	Postmortal & Stammzellen	Postmortal	OP-Präparate
Methode	Microarray	RNA-Seq	RNA-Seq	scRNA-Seq	scRNA-Seq	scRNA-Seq	RNA-Seq	Microarray	RNA-Seq
Methodisch homogen^a^	Nein	Nein	Ja	Nein	Nein	Nein	Nein	Ja	Ja
Komparative Analyse ^b^	Ja	Ja	Ja	Nein	Nein	Nein	Ja	Ja	Ja
Link	https://research.bioinformatics.udel.edu/iSyTE	https://sysbio.sitehost.iu.edu/express	http://retina.tigem.it	http://singlecell-eye.com	https://cells.ucsc.edu/?bp=eye	https://singlecell.broadinstitute.org	https://eyeIntegration.nei.nih.gov	https://genome.uiowa.edu/otdb	https://www.eye-transcriptome.com
Publikation	[12]	[5]	[19]	[29]	[25]	–	[26]	[30]	[33]

*AMD* altersabhängige Makuladegeneration, *BH* Bindehaut, *CNV* chorioidale Neovaskularisation, *FFPE* Formalin-fixiert und Paraffin-eingebettet, *ILM* Membrana limitans interna, *nAMD* neovaskuläre AMD, *PDR* proliferative diabetische Retinopathie, *PVR* proliferative Vitreoretinopathie, *RNA-Seq* RNA-Sequenzierung, *RPE* retinales Pigmentepithel, *scRNA-Seq* „single-cell RNA-Seq“, *TMW* Trabekelmaschenwerk, *UCSC* University of California, Santa Cruz

^a^Methodisch homogen, fasst folgende Qualitätskriterien zusammen: Bestätigung der histologischen Diagnose durch erfahrene Ophthalmopathologen, Verwendung des identischen Sequenzierungsprotokolls zur Minimierung der technischen Variabilität

^b^Komparative Analyse meint, dass alle Proben in ein gemeinsames bioinformatisches Modell eingeschlossen wurden, um so eine Normalisierung und damit Vergleichbarkeit der Expression zwischen verschiedenen Proben zu erreichen

### iSyTE und Express

Die Datenbanken *iSyTE* (https://research.bioinformatics.udel.edu/iSyTE) [12] und *Express* (https://sysbio.sitehost.iu.edu/express) [5] stellen die Transkriptionsprofile von Linsen- und Retinaproben der Maus zur Verfügung, wobei ein breites Spektrum an embryonalen und postnatalen Stadien enthalten ist. Somit werden eine intuitive Analyse und Visualisierung der Genexpression in verschiedenen Stadien der Linsen- und Netzhautentwicklung ermöglicht. Die Rohdaten stammen größtenteils aus öffentlich verfügbaren Datensätzen, welche durch unterschiedliche Sequenzierungsprotokolle an verschiedenen Institutionen generiert wurden, was die genannten Datenbanken durch eine methodische Inhomogenität limitiert. Zudem ist die Microarray-Technologie, auf der die *iSyTE*-Datenbank basiert, im Vergleich zur RNA-Sequenzierung durch eine höhere technische Variabilität sowie durch die fehlende Detektion von seltenen und neuen Transkripten limitiert [18]. Darüber hinaus können Microarray-Analysen nur diejenigen Transkripte nachweisen, für die eine entsprechende Sonde verfügbar ist, sodass es sich im Gegensatz zur RNA-Sequenzierung nicht um eine völlig unvoreingenommene Analyse handelt [18].

### retina.tigem.it

Die Datenbank *retina.tigem.it* (http://retina.tigem.it) enthält die Transkriptionsprofile von 50 gesunden humanen Netzhäuten, welche durch RNA-Sequenzierung methodisch homogen generiert wurden [19]. Somit wird ein umfangreicher und intuitiv durchsuchbarer Referenztranskriptomdatensatz der humanen Netzhaut angeboten. Bei den Proben handelt es sich um postmortales Gewebe, welches aufgrund der längeren Zeit zwischen Tod und Konservierung einem schnellen RNA-Abbau unterworfen ist, was die Daten in ihrer Aussagekraft beschränken kann [1, 22].

### Spectacle, UCSC Cell Browser und Single Cell Portal

Die Plattformen *Spectacle* (http://singlecell-eye.com), *UCSC Cell Browser* (https://cells.ucsc.edu/?bp=eye) und *Single Cell Portal *(https://singlecell.broadinstitute.org) ermöglichen die Exploration von umfangreichen Einzelzell-RNA-Sequenzierungsdaten von humanem Netzhaut‑, Aderhaut‑/RPE-, Kornea‑, Iris‑, Trabekelmaschenwerk- und Skleragewebe und enthalten zudem auch erkranktes Gewebe von Patienten mit Autoimmunretinopathie oder neovaskulärer AMD [29]. Der Anwender kann ohne bioinformatisches Hintergrundwissen analysieren, welche Zelltypen ein bestimmtes Gen exprimieren, welche Subpopulationen innerhalb eines Zelltyps vorliegen und kann zudem zelltypspezifische Markergene explorieren. Alle 3 Datenbanken basieren auf postmortalem Gewebe, sodass zuvor genannte Limitationen berücksichtigt werden müssen.

### Eye in a Disk

Die Datenbank *Eye in a Disk *(https://eyeIntegration.nei.nih.gov) ist mit einer Anzahl von 829 enthaltenen Proben die aktuell größte okuläre Transkriptomdatenbank [26], wobei verhältnismäßig wenig verschiedene Gewebearten (Retina, Aderhaut/RPE, Kornea, Linse) zur Verfügung stehen. Die Datenbank erlaubt als einzige einen Vergleich der okulären Transkriptionsprofile mit nichtokulären Geweben. *Eye in a Disk *ist durch postmortales oder aus Stammzellen gewonnenes Gewebe sowie durch methodische Inhomogenität limitiert.

### Ocular Tissue Database

Die* Ocular Tissue Database *(https://genome.uiowa.edu/otdb) stellt die Transkriptionsprofile einer mit 10 Entitäten verhältnismäßig großen Auswahl an verschiedenen gesunden humanen okulären Gewebearten zur Verfügung [30]. Die Datenbank enthält jedoch keine erkrankten okulären Entitäten und ist zudem durch die Microarray-Technologie und durch postmortal entnommenes Gewebe limitiert.

### Human Eye Transcriptome Atlas

**Figure 2:**
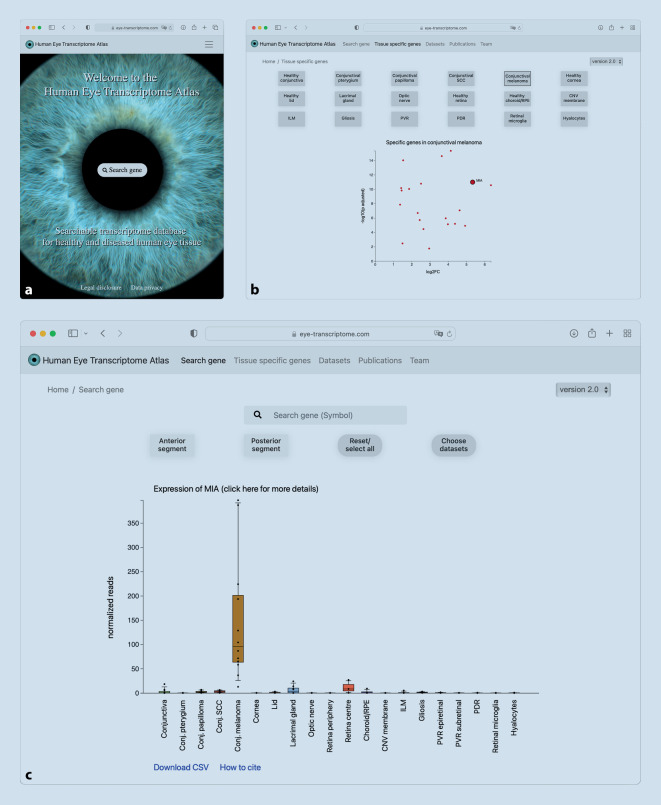

Der von unserer Arbeitsgruppe entwickelte *Human Eye Transcriptome Atlas* (https://www.eye-transcriptome.com, [33]) bietet unter den aktuell verfügbaren Datenbanken die größte Anzahl an verschiedenen okulären Gewebearten und enthält die meisten erkrankten okulären Entitäten – darunter Bindehautmelanome, Bindehautplattenepithelkarzinome, Bindehautpapillome, Pterygien sowie epiretinale Gliose, chorioidale Neovaskularisationsmembranen von Patienten mit neovaskulärer AMD, retinale Neovaskularisationsmembranen von Patienten mit proliferativer diabetischer Retinopathie und Membranen von Patienten mit proliferativer Vitreoretinopathie (epi- und subretinal) (Abb. 2). Mit insgesamt 139 Transkriptomdatensätzen gehört der *Human Eye Transcriptome Atlas* neben *Eye in a Disk *zu den beiden größten Datenbanken. Der *Human Eye Transcriptome Atlas *ist darüber hinaus die einzige Datenbank, die operativ entnommene Gewebeproben enthält, die unmittelbar nach der chirurgischen Entfernung entweder in RNA-Stabilisierungslösung überführt oder in Formalin fixiert und in Paraffin eingebettet (FFPE) wurden und anschließend sequenziert wurden [2, 4]. Dieses Vorgehen bietet den Vorteil, dass der schnelle RNA-Abbau, der bei postmortalen Proben auftritt, durch die unmittelbare Fixierung verringert wird [1, 22]. Alle Gewebeproben des *Human Eye Transcriptome Atlas *wurden an derselben Institution entnommen, prozessiert, durch erfahrene Ophthalmopathologen beurteilt und unter Anwendung desselben Sequenzierungsprotokolls sequenziert. Dies sichert einen hohen Qualitätsstandard der Proben und reduziert zudem die technische Variabilität der Sequenzierung.

## Fazit

Transkriptomdatenbanken wie der *Cancer Genome Atlas* [6] enthalten bisher kaum Augengewebe und erfordern für die Analyse der Sequenzierungsrohdaten spezielle bioinformatische Kenntnisse. Deshalb sind spezialisierte Datenbanken mit unterschiedlichem Anwendungsfokus entstanden, die Transkriptionsprofile okulärer Gewebe bereitstellen und gleichzeitig eine intuitive Datenanalyse ermöglichen. Unter den in dieser Arbeit zusammengefassten Datenbanken erlauben *Spectacle*, der *UCSC Cell Browser* und das *Single Cell Portal* des Broad Instituts eine intuitive Exploration von Einzelzell-RNA-Sequenzierungsdaten von Netzhaut‑, Aderhaut‑, Kornea‑, Iris‑, Trabekelmaschenwerk- und Skleragewebe. Der *Human Eye Transcriptome Atlas* bietet die größte Anzahl an verschiedenen Augengewebearten, enthält die meisten erkrankten okulären Entitäten und zeichnet sich durch einen hohen Qualitätsstandard aus, der durch methodische Homogenität erreicht wird. Okuläre Transkriptomdatenbanken bieten einen umfassenden und intuitiven Einblick in die Transkriptionsprofile verschiedener Augengewebe und -erkrankungen und ermöglichen somit eine unkomplizierte Hypothesenüberprüfung auf der Suche nach neuen diagnostischen und therapeutischen Zielen.
